# Habitat Suitability and Relative Abundance of the European Wildcat (*Felis silvestris*) in the Southeastern Part of Its Range

**DOI:** 10.3390/ani15192816

**Published:** 2025-09-26

**Authors:** Despina Migli, Christos Astaras, Nikolaos Kiamos, Stefanos Kyriakidis, Yorgos Mertzanis, George Boutsis, Nikolaos Oikonomakis, Yiannis Tsaknakis, Dionisios Youlatos

**Affiliations:** 1 Department of Zoology, School of Biology, Aristotle University of Thessaloniki, 54124 Thessaloniki, Greece; gboutsis@bio.auth.gr (G.B.); dyoul@bio.auth.gr (D.Y.); 2 Forest Research Institute, Hellenic Agricultural Organization “DEMETER”, 57006 Vasilika, Greece; 3 Natural History Museum of Crete, University of Crete, 71409 Heraklion, Greece; kiamosn@nhmc.uoc.gr; 4 CALLISTO Wildlife & Nature Conservation Society, 54640 Thessaloniki, Greece; kyriakidis@callisto.gr (S.K.); mertzanis@callisto.gr (Y.M.); gtsaknakis69@gmail.com (Y.T.); 5 School of Forestry and Natural Environment, Aristotle University of Thessaloniki, 54124 Thessaloniki, Greece; nick0981@gmail.com; 6 International Center for Biodiversity and Primate Conservation, Dali University, Dali 671003, China

**Keywords:** European wildcat, camera trapping, habitat model, Greece, occupancy, mesocarnivore

## Abstract

The European wildcat exhibits considerable plasticity in its habitat requirements across its distribution, with differences increasing along a continental-scale latitudinal gradient. While wildcats often favor deciduous and mixed forests with dense cover and prey, studies show these preferences vary across their expansion. Range-wide conservation efforts will benefit from incorporating knowledge generated by robust regional ecological models. We used data from a large camera trap grid (n = 292 stations), spanning across eight wildcat-associated habitats, within its range in northern Greece, to understand the regional ecological parameters affecting the species’ habitat selection. We analyzed the data using single-season density-induced detection heterogeneity occupancy models (Royle–Nichols), considering 12 environmental and anthropogenic parameters. The global model’s GoF was high (*p* = 0.9). Elevation and percent forest cover were both significantly negatively related to wildcat occupancy (as derived from the modeled “relative abundance index” N). Likewise, there was a negative, but moderate, relation between distance to freshwater bodies and human settlements with wildcat occupancy. We used the model-average coefficients to generate a predictive map of wildcat relative abundance across northern Greece, which identified 47,930 km^2^ of potential wildcat habitat. Assuming a range of densities between 0.05 and 0.3 ind/km^2^ in areas with predicted low, medium, and high relative abundance, we speculate the putative wildcat population in northern Greece to be between 3535 and 7070 individuals. The findings, which vary from ecological models of the species in northern Europe, show the need for regional models and the importance of Greece, and the Balkan peninsula, for the species.

## Simple Summary

The European wildcat exhibits varying habitat requirements across its distribution range, influenced by local environmental factors. Deciduous and mixed forests with dense undergrowth, which offer prey and seclusion from human activity, are often preferred habitats, yet studies indicate that such preferences vary across the species’ distribution. This study aimed to identify the primary determinants of wildcat habitat suitability of the species at the southeastern edge of the species’ range, in northern Greece, using camera trap data from eight sites (292 survey stations) and occupancy modeling. Our findings show that high-altitude forests are not favored by the species, unlike areas near water bodies and human settlements, which appear to be preferred. Overall, >47,900 km^2^ of suitable wildcat habitat occurs in northern Greece. Applying conservative density estimates for areas predicted to have low/medium/high putative wildcat densities, we speculate a northern Greece population of 3535 to 7070 individuals. The findings contribute to the broader understanding of the ecology, distribution, and conservation status of the European wildcat populations at the southeastern part of the species’ range, which remains, to date, understudied. Considering that the eastern Mediterranean region is among the fastest warming regions globally, understanding the ecological requirements of its fauna will help anticipate the conservation requirements of these species in the decades to come.

## 1. Introduction

During the last glacial period, European wildcat (*Felis silvestris*) populations survived in isolated refugia primarily located in broadleaved forests of the Mediterranean region, including the Iberian, Italian, and Balkan peninsulas [1,2]. These areas served as critical reservoirs from which the species gradually recolonized the rest of Europe. Today, five main biogeographic groups of European wildcats are recognized, inhabiting the Iberian Peninsula, Central Europe, Central Germany, the Italian Peninsula (including the island of Sicily), and the northeastern Italian and Balkan regions [3].

Although historically regarded as a forest specialist species [4,5,6], recent studies across these biogeographic groups indicate that the European wildcat exhibits remarkable ecological plasticity, enabling it to persist across a highly variable geographical and environmental gradient. For example, in Germany, the species is strongly associated with forested areas, particularly in proximity to forest edges and meadows [7,8,9], whereas in Scotland, wildcats have been observed using a broad spectrum of habitats, including coniferous and broadleaved woodlands, grasslands, and scrub patches [10,11]. In northern regions, the species’ range is limited by snow cover [12,13]. Meanwhile, in the Iberian Peninsula, scrubland habitats appear to be essential for wildcat persistence, with the presence of European rabbits (*Oryctolagus cuniculus*) playing a crucial role in determining local wildcat distribution [14,15,16].

Despite this adaptability, the European wildcat is a species of conservation concern in Europe, as it faces significant threats, leading to a fragmented and declining distribution across much of its range [8,15,17]. Habitat loss and fragmentation, hybridization with domestic cats, road mortality, and the emerging impacts of climate change are regarded as the most pressing challenges the wildcat faces. Habitat type and prey availability seem to be the main drivers defining the species’ expansion [11,18,19]. However, there is increasing evidence that local conditions play a key role in shaping its distribution, such as the presence of dense understory, where female wildcats, in particular, can shelter [7,20]. Spatial modeling has emerged as a valuable tool for assessing the environmental and anthropogenic determinants of wildcat distribution, providing insights that can inform conservation planning [16,21,22]. However, the reported ecological plasticity of the species dictates that habitat suitability models should be developed—or existing ones tested—in different regions.

Despite the growing body of research on European wildcat ecology, knowledge of the species’ habitat preferences in Balkan countries, including Greece, remains limited [23,24]. The wildcat populations in Greece are part of the “northeastern Italian and Balkan” biogeographic group and constitute the southeasternmost limit of the species’ distribution. Given the country’s diverse landscapes, ranging from mountainous forests to Mediterranean scrublands, it remains unclear which environmental factors influence wildcat presence and distribution in the region the most. In this study, we aim to address this knowledge gap by understanding drivers of wildcat habitat suitability across eight wildcat-associated habitats in northern Greece, using camera trapping data and occupancy modeling. We compare the findings to those from similar studies in other regions of the European wildcat range and present an estimate of the species population within the study area. This approach allows us to address two fundamental questions for the species’ conservation—where populations are located and how many individuals they comprise.

The improved understanding of the ecological requirements of the European wildcat in the region may help anticipate the ecological requirements of the species in the decades to come and develop tailored conservation measures. This would be especially useful considering that eastern Mediterranean-region wildlife is among the most vulnerable to the impacts of global warming [25,26] and that wildcats of the Mediterranean biome are among the most threatened by hybridization [27]—a key threat to the species that can be better addressed through an improved understanding of its ecological requirements and population dynamics.

## 2. Materials and Methods

### 2.1. Study Area

**Figure 1:**
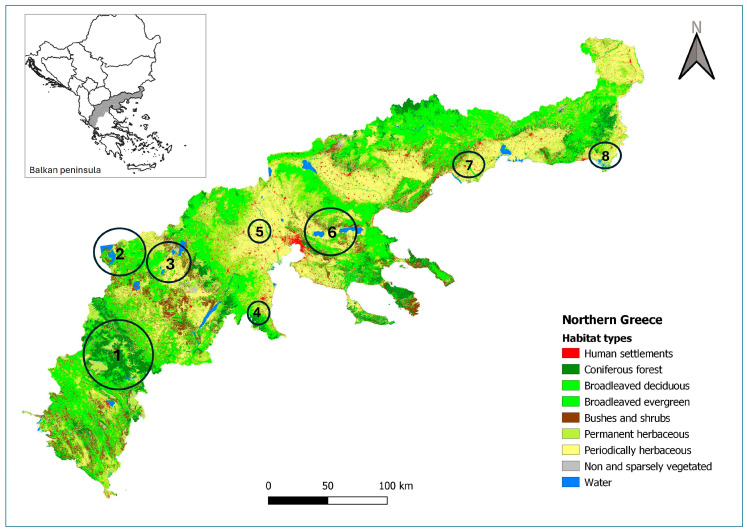
Map depicting the habitat types of the study area and the location of the camera trapping sites. 1: Pindus Mt (82 camera traps); 2: Prespa Lake (62 camera traps); 3. Amyntaio–Kozani area (42 camera traps); 4. Olympus Mt (6 camera traps); 5. Axios River (9 camera traps); 6. Koronia and Volvi Lakes National Park (64 camera traps); 7. Nestos River (17 camera traps); 8. Evros Delta (10 camera traps).

The study covers an area of 50,350 km^2^ in northern Greece, encompassing the regions of Macedonia, Thrace, and Epirus. The area is characterized by a typical Mediterranean climate, where annual rainfall occurs almost entirely during the winter, and summer droughts are common. There are distinct variations in topography and land use. The region’s western part is more mountainous (mean elevation = 938 m [28]), featuring high precipitation levels (1000–1500 mm per annum [29]) and extensive coniferous and broadleaved forests (Figure S1). In contrast, the eastern part consists of lower mountain reliefs (mean elevation = 332 m [28]) and is predominantly utilized for agriculture and livestock grazing (Figure 1). The area includes nine national parks, contributing to its conservation significance. Vegetation cover is dominated by broadleaved forests, which account for 36.4% of the total area. These forests primarily consist of Downy oak (*Quercus pubescens*), Hungarian oak (*Quercus frainetto*), and European beech (*Fagus sylvatica*). Coniferous woodlands, comprising species such as fir (*Abies* spp.), black pine (*Pinus nigra*), and Aleppo/Calabrian pine (*Pinus halepensis/brutia*), cover 8.2% of the region. Other land cover types include grasslands, reeds, and meadows (22.7%); arable land (16.75%); and bushes and shrubs (9.3%). The region is rich in hydrological features, including numerous rivers, small watercourses, and lakes. Lowland wetlands are primarily concentrated in the eastern part of the study area, whereas water bodies in the west are generally located above 200 m altitude. Human density in northern Greece is moderate, with an average of 49 persons per km^2^ [30]. The region has a medium-density road network (>90 km per 100 km^2^) and is traversed along an east–west axis by one of Greece’s major motorways, the Egnatia Odos, which facilitates transportation and connectivity across the study area.

### 2.2. Data Collection and Camera Trapping

Data collection was conducted over a six-year period, from 2015 to 2021. A total of 292 camera traps were deployed across eight selected survey sites in northern Greece (Figure 1), representing most potential wildcat habitat types. At some sites, the camera trap survey grid was completed by redeploying camera traps at additional stations, but always in consecutive months (not across multiple years). Deployments occurred from November to April, which coincides with the wildcat’s breeding season. All survey sites included—at least in part—Natura 2000 sites (which are included in the European Union’s ecological network of protected sites established to protect species and habitats) [31,32]. High-altitude mountainous habitats were represented by the Pindus Mountain (39°59′ N, 21°0′ E) and Olympus Mountain (40°7′ N, 22°23′ E) survey sites, where 82 and 6 camera traps were installed, respectively, in coniferous and broadleaved woodlands. Highland wetlands above 900 m were included in the Prespes Lakes survey site (40°47′ N, 21°4′ E), surrounded primarily by broadleaved deciduous forests and maquis vegetation. In this area, 62 camera traps were deployed. Lowland wetland ecosystems were included in the Evros Delta (40°47′ N, 26°3′ E), Nestos River (40°53′ N, 24°46′ E), and Axios River (40°36′ N, 22°42′ E) survey sites, where a total of 36 camera traps were placed. The predominant habitats on these sites are marshlands, meadows, and arable land. Finally, the Amyntaio site (40°40′ N, 21°35′ E) and the Koronia–Volvi Lakes National Park (40°39′ N, 23°16′ E) encompass a mosaic of diverse habitats and altitudinal ranges from 30 m to 1000 m, where 43 and 63 camera traps were installed, respectively.

In each survey site, we deployed motion-activated camera traps within a 2 × 2 km grid, reflecting the mean home range of a female European wildcat. The location of the camera within each grid cell was not random but rather selected to represent the dominant habitat type of the grid cell. Moreover, cameras were mounted on tree trunks at approximately 50–60 cm height, oriented toward unpaved forestry roads or hiking trails to maximize the likelihood of detecting elusive species, including the European wildcat. Two camera models were used—Browning Dark Ops (Birmingham, Alabama, USA) and Bushnell Core DS (Overland Park, Kansas, USA). Both models were equipped with no-glow infrared LED flash and programmed to record a single 20 s video upon activation, with a 0.2 s delay between successive triggers. Within each of the eight sites, the same model of camera traps was used. Each station remained active for a minimum of 30 consecutive days and was checked every 10–15 days for battery replacement and SD card retrieval. The captured footage was manually processed and annotated at the species level. Wild-living felids were classified as putative wildcats based on pelage characteristics and coat patterns, following the criteria established by Kitchener et al. [33] and Ragni and Possenti [34]. The classification approach proposed by Sforzi and Lapini [35] was applied, retaining only images that met C2 (wild phenotype, not confirmed by other methods) and C3 (partially visible or difficult-to-interpret phenotype with wild traits) criteria for further analysis. It is noted that hereafter, all wildcat references and results within this study account for putative wildcats. To avoid inter-observer bias, all felid classification processes were performed by a single observer (DM) who has >10 years of experience in small felid identifications in the study area (field observations, taxidermy collections, and trapping and handling feral, hybrid, and wildcats). Consecutive wildcat photos within a 30 min interval were considered as part of the same independent detection. Following this screening, the site and date of all putative wildcat detections were extracted from the full dataset and used to construct the matrix for occupancy analysis.

### 2.3. Statistical Analysis

#### 2.3.1. Variables

We evaluated the influence of three anthropogenic and nine environmental variables on wildcat occupancy and/or detection (Table 1). Anthropogenic factors included road length, distance to human settlements, and population density. Environmental covariates encompassed forest edge density, vegetation metrics based on the Plant Phenology Index elevation, slope, distance to major water bodies, and land cover types. Variable selection was guided by the key ecological requirements of the wildcat, namely prey availability, shelter, and sensitivity to human disturbance [6,8,18,21,36,37]. The four primary land cover variables analyzed were (1) general forest cover (including small woody patches), (2) coniferous woodland, (3) mixed and broadleaved woodland, and (4) open fields, which comprised meadows and agricultural areas. Coniferous and broadleaved woodlands were treated separately due to their potential differences in prey abundance and understory complexity. Open fields were included due to their known association with increased wildcat prey availability, particularly small rodents such as mice and voles. Human disturbance—known to constrain wildcat distribution [38,39]—was assessed through the spatial extent of urban settlements and road infrastructure. Forest edge density was used as a proxy for landscape heterogeneity. This was calculated using a matrix-based approach, assigning differential weights to edge types, thereby capturing a more nuanced, gradient-based transition between habitats. Elevation was included as a potential proxy for seasonal habitat use, enabling vertical movements that may support wildcat persistence during periods of environmental stress (e.g., winter). Finally, the presence of large water bodies (e.g., lakes and rivers) was also considered, given their recognized importance for wildcat survival and dispersal [8].

#### 2.3.2. Occupancy Modeling

We fit likelihood-based single-season occupancy models to estimate the probability of wildcat occurrences, accounting for imperfect detection using R v 4.4.2 [47] and the ‘unmarked’ package [48]. We treated each camera trap station as a site. Since occupancy analysis requires multiple sampling occasions to estimate the detection probability of a species, we summarized wildcat detection (1) or non-detection (0) for each site into 5-day detection windows (i.e., temporal replication). Considering the species’ spatial ecology in northern Greece [49], the detection window used was considered appropriate for increasing the temporal independence of detections and reducing data overdispersion. Moreover, since all camera traps operated for no more than two months, we considered the population closure assumption of the single-season model as being met.

Using information–theoretic approaches and the Akaike Information Criterion (AIC) for model selection [50], we compared baseline (intercept only) single-season MacKenzie [51] and Royle–Nichols [52] models. There was strong support for using the latter, so we only considered the Royle–Nichols model in further analysis. The model uses a Poisson distribution and allows for heterogeneity in a species’ detection probability due to differences in its abundance at each site. The modeled parameters are expected site abundance (N) and probability of detecting an individual of the species (r), with occupancy (psi) and overall species detection probability (p) being derived parameters. We considered a series of environmental and anthropogenic parameters of wildcat expected site abundance (N) (Table 1) by calculating their mean value within a 500 m radius from each camera trap (station)—a scale which reflected the minimum home range of a female European wildcat. Parameter values were standardized (z-transformation) to help stabilize the numerical optimization algorithm of the models [53].

We proceeded with a three-step model-building approach designed to reduce excessive model testing and to manage model complexity and therefore possible overfitting. First, we assessed the parameters that could affect detection probability—namely camera trap grid (site) and camera trap operation duration—while keeping the state process (N) of the hierarchical model constant (intercept only) across sites (as per [54]). Second, we selected an optimal set of informative expected site abundance (N) parameters by running univariate models and comparing their AIC against that of the null (intercept only) model, while keeping detection (r) fixed according to the best parameterization of the first step. We checked for collinearity (Pearson’s correlation |r| > 0.7) among all informative expected site abundance (N) parameters and kept from each correlated pair the parameter with the lowest univariate model AIC [55]. Finally, we considered all combinations of the final set of informative parameters as being equally biologically plausible to describe the drivers of wildcat site-level abundance. To do this, we developed a multivariate model with all abundance and detection parameters (global model) and used the “dredge” function of the MuMIn R package Version 1.47.5. [56] to run all parameter combinations. Since the AIC weight of the best model was low (<0.85), we calculated the model average beta coefficients of all models with cumulative AIC weight = 0.95 [57] using the “model.avg function” of the AICcmodavg package (Mazerolle 2023). We tested the global model’s goodness of fit with a Mackenzie–Bailey’s goodness of fit test, which uses Pearson’s chi-square statistics (function mb.gof.test:AICcmodavg). We generated a putative wildcat relative abundance map for the study area using the conditional model-averaged beta coefficients [58]. The map was used both to estimate the species range in northern Greece and to speculate—conservatively—about the wildcat population. European population densities of the species exhibit considerable variation across the species’ range, from as low as 0.1 to as high as 250 individuals per 100 km^2^, depending on regional conditions and methodological differences [59]. Due to the absence of direct field-based density estimates for the study area, we adopted an approach analogous to that used by the IUCN for estimating European wildcat populations, which typically rely on conservative density values [59]. Specifically, relative abundance values generated by the model were reclassified into four categories—absent, low, medium, and high. The classification thresholds of the relative abundance categories were unavoidably subjective and were the result of trials intended to capture the perceived variation in the natural densities of the species in the Greek countryside based on our many years of experience studying the species. To translate these classes into density estimates, we assigned a conservative range of values (individuals/km^2^) to each category—0.05–0.1 for low, 0.1–0.2 for medium, and 0.15–0.3 for high—keeping values even at the high density class close to the lower bounds reported for the species across its range. We then multiplied the area of each wildcat density class to speculate a lower and upper range of putative wildcat population size in northern Greece, grounded in habitat suitability patterns. Our estimates should be interpreted only as a first approximation to guide conservation planning, rather than definitive population numbers.

## 3. Results

The total camera trap survey effort was 10,911 trap nights (mean 1364 ± 1108 SD per site; range 244–3253), and the total average deployment duration was 37.4 days, resulting in 319 wildcat detections (mean 39.9 ± 30.8 SD per site; range 8–88) (Table 2). The site with the highest and lowest wildcat encounter rate was Amyntaio and Pindos with 5.7 and 1.7 detections per 100 camera trap nights, respectively. The proportion of camera traps with at least one wildcat detection (naïve occupancy) ranged from 0.23 in Prespes Lakes and Pindos Mts. to 1 in Olympus Mt.

Because of the need to rotate cameras in the larger grids and the consequent relatively low number of trapping nights per station, detection probabilities may have been reduced, increasing uncertainty in relative site abundance estimates (N).

### Wildcat Abundance

**Figure 2:**
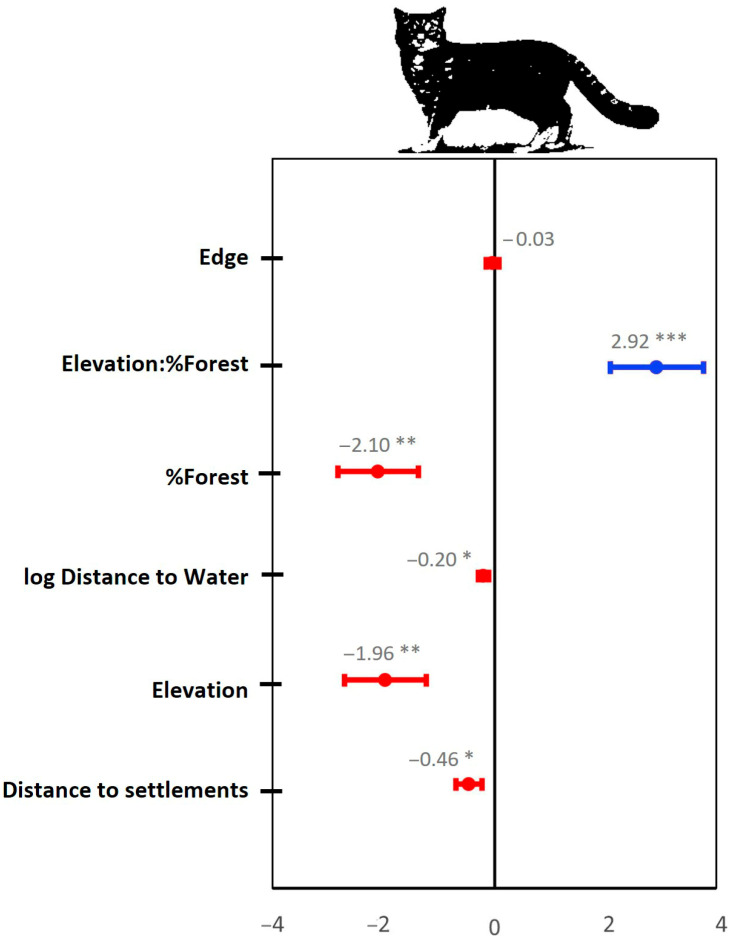
Conditional model-averaged beta coefficient estimates and 95% CIs of covariates describing expected wildcat site abundance (N) (Royle–Nichols model). Asterisks denote significance at the 0.05 ( * ), 0.01 ( ** ) and 0.001 ( *** ) level.

There was strong support for the inclusion of site as a wildcat detection probability covariate, so it was included in all subsequent Royle–Nichols models considered. Univariate models of expected wildcat site abundance (N) identified elevation, log distance to water, distance to settlement, forest edge, percent forest cover, slope, percent broadleaf forest cover, and percent farmland cover as being informative of wildcat relative abundance (i.e., univariate model AIC < null model AIC, Table S1). The latter three covariates were excluded from the global model to avoid multicollinearity, as they were highly correlated with more informative covariates (Table S2). In the global model, we included a pair-wise interaction for elevation and percent forest cover, as it substantially improved model performance and matched our field observations for the species. The global model demonstrated a satisfactory goodness of fit (χ^2^ test, *p* = 0.925), indicating strong agreement between the model and the observed data. All covariates included in the model average, except for forest edge density, had a significant effect on the predicted expected site abundance (N) of wildcats (Figure 2).

**Figure 3:**
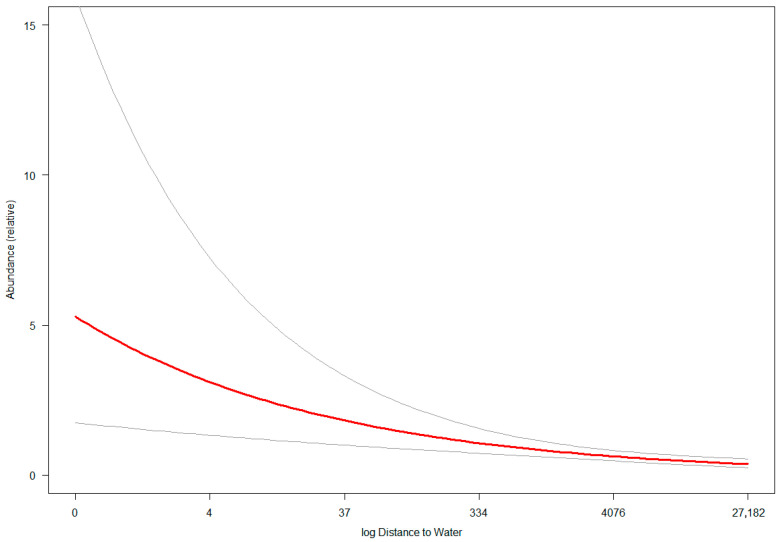
Response (red) and 95% confidence interval (gray) curves of wildcat relative abundance to log distance to water.

**Figure 4:**
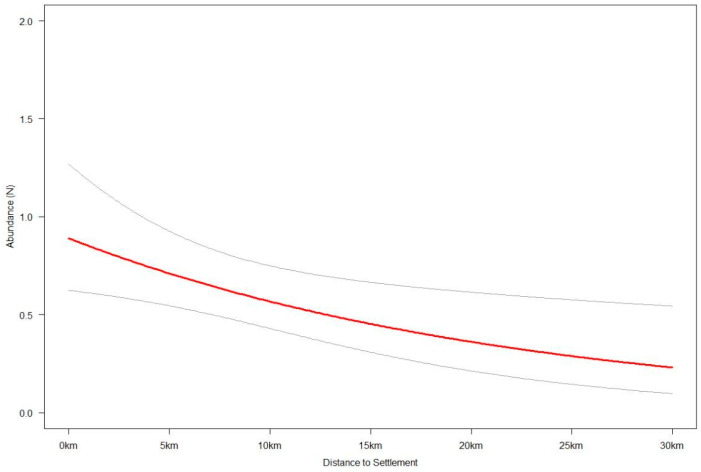
Response (red) and 95% confidence interval (gray) curves of wildcat relative abundance to distance from human settlements.

**Figure 5:**
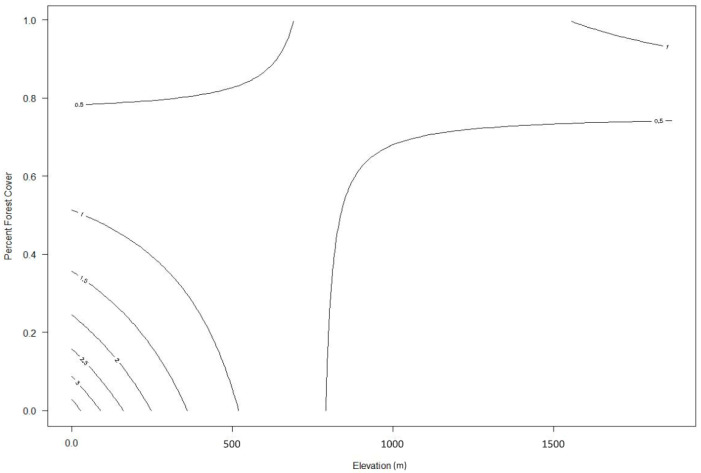
Contour plot showing the predicted wildcat relative abundance along a gradient of elevation and percent forest cover.

Specifically, wildcat relative abundance declines sharply as distance to water sources (lakes and rivers) increases, with this effect becoming negligible for distances beyond 300–400 m (Figure 3). The large confidence intervals at shorter distances show that there is high variability in this effect. Wildcat relative abundance also decreases as distance to settlements increases, with this slight negative effect having a linear and stable relationship across the range of distances observed in our study area (Figure 4). However, the increase in wildcat relative abundance in areas closer to human settlements is not as pronounced as that for areas closer to water sources. In terms of elevation and percent forest cover, wildcat relative abundance is highest in areas with low-to-middle elevation (0–500 m) and low-to-medium forest cover (0–40%). Sparsely forested lowland areas have up to seven times higher wildcat density than densely forested low-to-mid-elevation (<600 m) areas or higher-elevation (>700 m) areas with forest cover between 0 and 40% (Figure 5). Areas above 800 m have relatively low wildcat relative abundance regardless of percent forest cover. Similarly, areas with high percent forest cover (>80%) have low wildcat density regardless of elevation.

**Figure 6:**
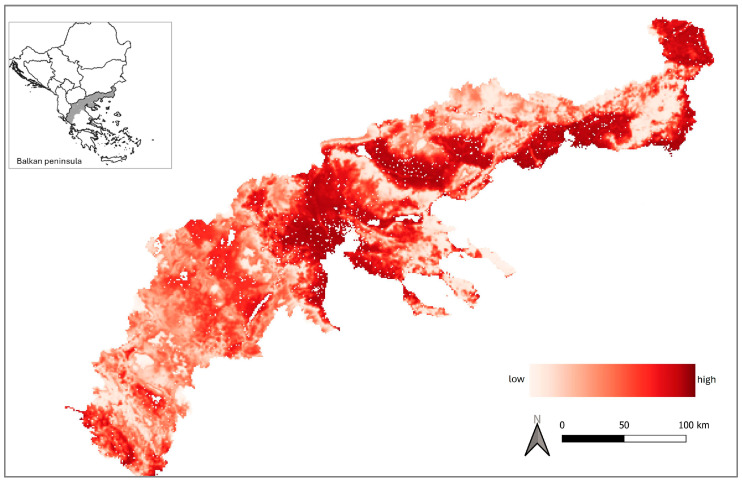
Predictive map of European wildcat habitat suitability (scaled relative abundance to 0–1 range) across northern Greece. Areas that by default cannot host wildcats (urban areas, lakes, and rivers) are depicted in white.

We used the covariates’ conditional model-averaged beta coefficients to generate a predictive map of European wildcat relative abundance across northern Greece (Figure 6). The potential range for the species (abundance > 0) is 47,930 km^2^, of which 11.2% (5410 km^2^) has the highest suitability (relative abundance). Low-suitability areas account for >62% of the potential wildcat range and are most prevalent in the mountainous western part of the study area.

**Figure 7:**
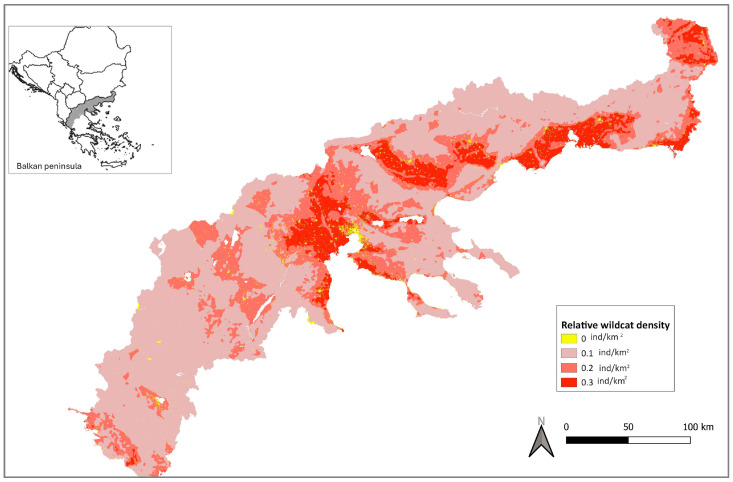
Map of European wildcat relative density across northern Greece used to speculate the putative population of the species.

The relative abundance values ranged from 0 to 62. We classified areas with no wildcats, and low-, medium- and high-density wildcats using, respectively, 0–0.0001, ≥0.0001–1.5, ≥1.5–20, and ≥20–62 thresholds. Applying a range of density values to the above areas, we speculate northern Greece to have between 3535 and 7070 (Table 3, Figure 7) putative wildcats.

## 4. Discussion

Our findings indicate that across northern Greece, European wildcat densities are highest in low-elevation, sparsely forested areas, near water sources, and human settlements, declining markedly at higher-elevation forested landscapes. Wildcats do not strongly avoid human-dominated areas and, in some contexts, may even tolerate or utilize habitats relatively close to settlements, possibly due to factors like prey availability or edge habitats. However, the lack of a sharp increase in relative abundance near settlements indicates that human areas do not provide strong benefits, pointing to potential coexistence in shared landscapes.

The observed dissimilarity of our habitat suitability results to those reported for wildcats in Central and Northern Europe (e.g., [8,12,60]), where the species is associated with dense forests, as well as the similarity to those from Mediterranean regions with comparable land cover and climatic conditions (e.g., [18,61,62]), were anticipated. However, our results contrast with studies from the Iberian and Italian Peninsulas. In northwestern Spain, for instance, Vázquez García et al. [16] reported a positive association between wildcat presence and both elevation and forest cover, coupled with the avoidance of areas with high footpath density, suggesting sensitivity to human disturbance. Similarly, in Sicily, wildcat occupancy was positively associated with mixed forest landscapes [63], while in eastern Portugal, the species showed a preference for mountainous terrain [64]. Only in central Spain, wildcats reportedly avoid dense forests and higher elevations [61], akin to what we report for wildcats in northern Greece. In both central Spain and Portugal, wildcat abundance has been strongly linked to scrub–pasture mosaics rich in rabbits [21,64], underscoring the role of lagomorph availability as a key determinant of wildcat habitat suitability. In Greece, where rabbits are absent within the wildcat range, the importance of scrublands was not supported. The most notable divergence from previously reported wildcat habitat preferences is the apparent tolerance to human proximity observed in our study, whereas the avoidance of human disturbance has generally been considered universal [16,35,55]. This pattern is likely associated with the higher prey availability found at the interface between agricultural and natural habitats [34,56,57], which can support larger wildcat populations than prey-poor areas. Moreover, in northern Greece, the prevalent low-to-medium-intensity agricultural practices create diverse, well-connected landscapes that are generally favored by wildcats [18]. The combination of greater prey abundance and a mosaic-structured agricultural matrix appears to buffer the influence of human settlements, which are widely scattered within this landscape. In contrast, the observed positive relation of wildcat relative abundance and proximity to water sources is in accordance with findings of previous studies across the species’ range (e.g., [8,11,65]). Collectively, the present and previous wildcat habitat suitability studies emphasize the ecological flexibility of the European wildcat, which appears to respond to regional variations in prey availability, levels of human activity, and habitat structure. 

The speculated population size of putative wildcats in northern Greece, even based on conservative model assumptions, could range between 3535 and 7070 individuals. This figure is relatively high compared to estimates from other European countries. For instance, the German wildcat population is reported to be approximately 5000 to 7000 individuals (Article 17 species assessments are evaluations of the conservation status of species listed in the EU Habitats Directive, required every six years from Member States to monitor progress toward biodiversity goals [66]), with an additional 1100 individuals in neighboring Switzerland [12]. In the Balkans, Croatia’s wildcat population is estimated to number less than 2000 individuals [67], Bulgaria’s less than 5000, and Romania’s, where the species is widespread, between 8005 and 9150 individuals [66].

Several factors may account for the comparatively high population estimate in northern Greece. In Central Europe, wildcat populations experienced severe declines in the first half of the 20th century due to extensive habitat loss and intense persecution [4,68]. Recovery only began following legal protection measures introduced in the second half of the century. In contrast, the Greek landscape appears to have followed a different historical trajectory. Deforestation, including large-scale pinewood loss, occurred approximately 2000 years BCE [69], and more recent landscape dynamics show a reduction in grasslands, open shrublands, and silvopastoral areas over the last 75 years, in favor of denser shrubland and forest cover [70]. Moreover, the Balkan Peninsula served as a key glacial refugium for the species during the Last Glacial Maximum [3,71], resulting in the longer continuous presence of the species in the region and—potentially—higher levels of adaptation to its climatic conditions.

Moreover, harsh winter conditions, particularly prolonged snow cover, have been shown to significantly influence wildcat distribution in Central Europe. Studies have indicated that the presence of snow exceeding 20 cm in depth for more than 100 consecutive days can severely restrict wildcat mobility and reduce hunting success, as prey species tend to remain hidden beneath the snow [72,73]. Such conditions, however, are relatively rare and localized in Greece. Persistent snow cover is largely confined to high-altitude zones in the mountainous regions of northern and central Greece, particularly along the Pindus Mts. range and near the northern borders with Albania and North Macedonia. Furthermore, recent climatic trends show a marked decline in snow cover across Greece over the 1991–2020 period [74]. Given these climatic conditions and the wildcat’s general avoidance of high-altitude environments, snow cover appears to play a minimal role in shaping the species’ distribution in Greece. This further supports the notion that the Greek landscape, particularly in its lower elevation zones, offers comparatively favorable and stable habitat conditions for wildcat populations.

In addition, historically, wildcat persecution in Greece has been low, compared to many other regions where felids were actively targeted. Culling focused mainly on canids (especially golden jackals, red foxes, and wolves) [75]. The combination of these ecological, historical, and sociocultural factors likely resulted in a continuous and relatively stable wildcat population, which could explain the reported present high population estimate for the northern part of the country.

It is broadly acknowledged that studies employing camera trapping for population density estimates often refer to putative European wildcats, rather than genetically verified individuals. As a result, individuals with wild phenotypes but hybrid ancestry are likely to be included in abundance estimates. Hybridization between European wildcats and domestic cats (*Felis catus*) is a well-documented conservation concern, with genetically confirmed hybrids reported in most European regions where hybridization has been assessed. In Greece, however, published data on hybridization levels remain limited or entirely absent [76]. Unpublished research conducted by the primary authors of this study indicates a relatively high hybridization rate in northern Greece, reaching approximately 19.8%. This finding aligns with data from neighboring Balkan countries, where hybridization rates between wild and domestic cats range from 13% to 52%, as reported in Slovenia, Croatia, Serbia, and North Macedonia [67]. The relative density map presented in this study can be used to identify priority areas for hybridization mitigation measures (e.g., neutering campaigns). Given the conservation implications of introgression, it is strongly recommended that future population assessments of European wildcats be accompanied by genetic analyses. This integrative approach would provide a more accurate and comprehensive understanding of the species’ status.

Climate change presents a significant and escalating threat to biodiversity, with wide-ranging effects across ecological networks and trophic levels. While felids are generally among the less-studied carnivore families in terms of climate sensitivity, existing research suggests that many mesocarnivores are likely to face range contractions under future climate scenarios [77]. In the Mediterranean Basin, climate projections until the end of the century consistently forecast an increased frequency and intensity of droughts and heatwaves and decreased total annual precipitation [78], with water scarcity becoming more acute, especially during the summer months [79]. Water stress combined with altered disturbance regimes (especially wildfire frequency) are expected to amplify the impact of climate change on Mediterranean-type ecosystems [80], significantly affecting local vegetation dynamics, particularly in low-elevation areas where dry-adapted species are likely to proliferate, altering current plant community structure. Since low-elevation areas have the highest wildcat relative abundance in our study and the species does not prefer shrubland, the anticipated vegetation changes are likely to lead to significant range contraction for the European wildcat, especially if water-dependent prey species also decline. For the above reasons, it is reasonable to expect that the reported current favorable population status of the European wildcat in northern Greece will change in the decades ahead due to climate change; therefore, a robust monitoring protocol should be established for the species.

## 5. Conclusions

This study provides the first spatially explicit relative abundance model of the European wildcat (*Felis silvestris*) in northern Greece, offering crucial insights on the habitat suitability and potential distribution of the species in a region with previously limited published information. Our findings identify low-elevation, sparsely forested areas near water sources, as well as human settlements, as key habitats for the wildcat, with the species’ relative abundance declining sharply as elevation and dense forest cover increase. The predicted wildcat population of 3535–7070 individuals is higher than that of most European countries. However, it includes all putative wildcats based on their phenotype due to the inability of genetically validating individuals from camera trap images.

Our results also underscore the broader implications of climate change, land use history, and human–wildlife interactions in shaping wildcat distribution patterns in Mediterranean ecosystems. Unlike most Central and Western European populations, wildcats in northern Greece appear to thrive in human-modified landscapes, possibly due to favorable prey dynamics in agro-natural mosaics. However, expected climate changes, including rising temperatures, water scarcity, and increased fire frequency, are anticipated to significantly alter the habitat of the lowland areas where wildcats are most abundant today. Considering that threats from hybridization and habitat degradation are also present, it becomes apparent that wildcat conservation measures are needed to secure the long-term persistence of the species in this southeastern European stronghold, combining habitat protection, genetic monitoring, and climate-adaptive management.

## Figures and Tables

**Table 1:** Site covariates considered in the European wildcat occupancy modeling.

Variable	Description	Source
Road length	Total road length within a 500 m radius area	OpenStreetmaps [40]
Forest edge	Mean value within a 500 m radius area of the total edge (m) between forest and non-forests in a radius of 100 m	FragStats 4.2.1 Cross Weighed Edge Density
Plant Phenology Index	Mean value within a 500 m radius area of the derived linear trend (2000–2016) for above-ground plant productivity	European Environment Agency [41]
Elevation	Mean elevation value within a 500 m radius area	Copernicus EU (EU-DEM v1.1) [42]
Slope	Mean slope value within a 500 m radius area	Derived from Elevation using QGIS v.3.16.14
Distance to water	Mean Euclidean distance to the nearest source of water within a 500 m radius area	Copernicus EU (CLCplus Backbone) [43]
Distance to settlement	Mean Euclidean distance to the nearest human settlement within a 500 m radius area	Geodata.gov.gr + QGIS v.3.16.14 [44]
Percent forest cover	Percent of all forest type cover within a 500 m radius area	Copernicus EU (CLC + SmallWoodyFeatures) [43,45]
Percent conifer forest cover	Percent of pine forest cover within a 500 m radius area	Copernicus EU (CLCplus Backbone) [43]
Percent broadleaf forest cover	Percent of broadleaf forest cover within a 500 m radius area	Copernicus EU (CLCplus Backbone) [43]
Percent farmland cover	Percent of agricultural field cover within a 500 m radius area	Copernicus EU (CLCplus Backbone) [43]
Population density	Mean human population density within a 500 m radius area	Eurostat [46]

**Table 2:** Survey effort, wildcat detections, and naïve occupancy per site.

Sites	Survey Effort (Trap Nights)	Camera Traps	Survey Period	Deployment Duration (Mean ± SD Days)	Wildcat Detections *	Encounter Rate (per 100 Trap Nights)	Naïve Occupancy **
Evros	244	10	03–04/2015	24.4 ± 6.1	8	3.3	0.70
Nestos	528	17	01–04/2015	31.1 ± 2.6	17	3.2	0.53
Koronia–Volvi	2231	64	11/2015–04/2016	35.4 ± 6.7	70	3.1	0.31
Axios	230	9	02–03/2016	25.6 ± 7.2	6	2.6	0.44
Amyntaio	1535	42	11/2016–04/2017	35.7 ± 7.2	88	5.7	0.57
Olympos	390	6	01–03/2021	65 ± 0	11	2.8	1.00
Prespes	2500	62	11/2020–04/2021	40.3 ± 12.4	65	2.6	0.23
Pindos	3253	82	11/2020–04/2021	39.7 ± 13.1	54	1.7	0.23

* Independent detections (≥30 min interval between subsequent wildcat detections). ** Proportion of camera traps with at least one wildcat detection.

**Table 3:** Classification rules for linking the relative abundance categories to relative density classes.

Classification Thresholds (Range of N Values)	Area (km^2^)	Density (ind/km^2^)	Individuals
Absent (0–0.0001)	1055	0	0
Low (≥0.0001–1.5)	30,580	0.05–0.1	1529–3058
Medium (≥1.5–20)	11,945	0.1–0.2	1194–2389
High (≥20–62)	5410	0.15–0.3	811–1623

## Data Availability

The data presented in this study are available on request from the corresponding author, as a portion of the dataset is part of ongoing research and unpublished analysis, and any revelation of the exact placement could jeopardize the deployed equipment.

## Supplementary Materials

The following supporting information can be downloaded at https://www.mdpi.com/article/10.3390/ani15192816/s1, Figure S1: (a) Altitude range and (b) average annual precipitation in Greece. Table S1: Model selection of univariate expected site abundance models, compared to the null (intercept only) model for expected wildcat site abundance. (Note: All models contain Site as a covariate of wildcat detection probability r). Table S2: (a) Correlation matrix of considered parameters and (b) AIC of univariate model results.
