# Supplementary material for: Habitat Suitability and Relative Abundance of the European Wildcat (Felis silvestris) in the Southeastern Part of Its Range
Source: Animals (Basel). 2025 Sep 26;15(19):2816. doi: 10.3390/ani15192816 (PMC12524287; doi:10.3390/ani15192816)
Supplement: Supplementary file 1 [file animals-15-02816-s001.zip › animals-3816956-supplementary.pdf]

Figure S1. (a) Altitude range and (b) Average annual precipitation in Greece

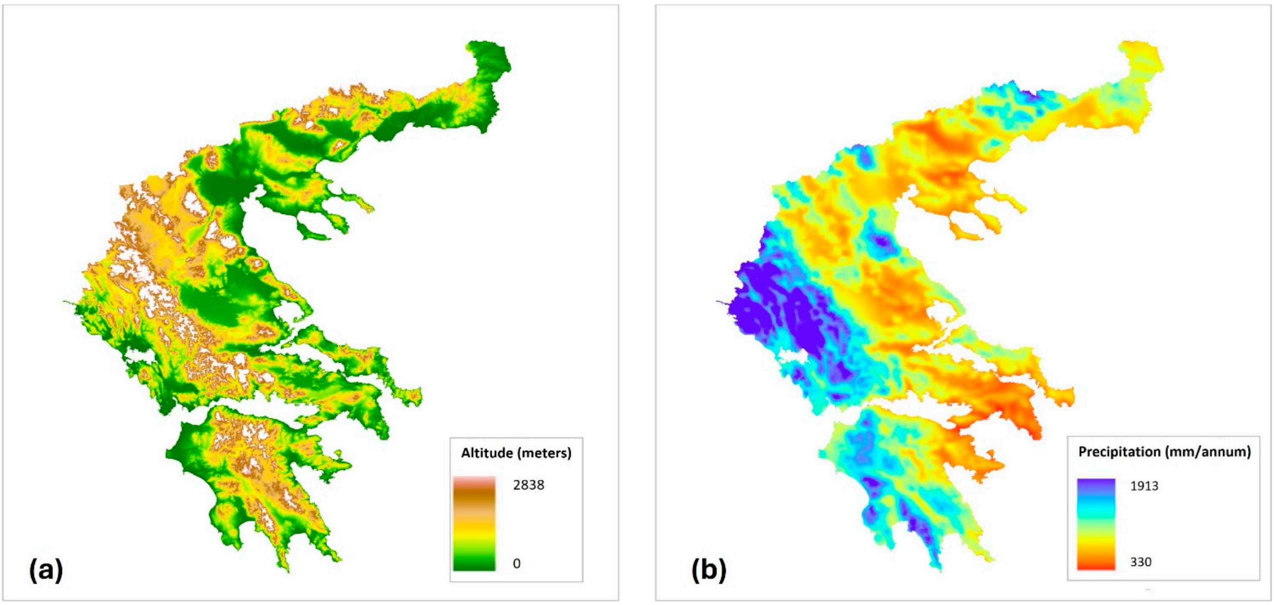

Table S1. Model selection of univariate expected site abundance models, compared to the null (intercept only) model for expected wildcat site abundance. ([Note: All models contain Site as a covariate of wildcat detection probability r).

**Model**	**AIC**	**ΔAIC**
Null+Elevation	1236.715	0
Null+Distance to water	1244.603	7.888
Null+Slope	1249.317	12.602
Null+Percent broadleaf forest cover	1251.37	14.655
Null+Distance to settlement	1251.684	14.969
Null+Percent forest cover	1252.675	15.96
Null+Percent farmland cover	1253.723	17.008
Null+Forest Edge	1253.825	17.11
**Null**	**1256.238**	**19.523**
Null+Plant Phenology Index	1256.516	19.801
Null+Population density	1256.905	20.19
Null+Road Length	1257.923	21.208
Null+Percent conifer forest cover	1258.2	21.485

Table S2. (a) **C**orrelation matrix of considered parameters and (b) AIC of univariate model results

(a)

	Edge	Elevation	Slope	Percent Forest Cover	log Distance to Water	Percent Broadleaf Forest Cover	Percent Farmland Cover	Distance to human settlement
**Edge**								
**Elevation**	-0.045							
**Slope**	-0.119	**0.789**						
**Percent Forest Cover**	-0.059	0.466	0.668					
**log Distance to Water**	0.194	0.584	0.664	0.625				
**Percent Broadleaf Forest Cover**	0.032	0.242	0.416	**0.751**	0.355			
**Percent Farmland Cover**	-0.034	-0.613	**-0.694**	**-0.700**	-0.412	-0.558		
**Distance to human settlement**	-0.044	0.326	0.111	-0.039	-0.282	0.105	-0.179	

(b)

UNIVARIATE MODEL RESULTS	AIC
log Distance to Water	1273.5
Slope	1277.3
Elevation	1277.8
Distance to human settlement	1282.8
Percent Forest Cover	1283.0
Edge	1283.5
Percent Farmland Cover	1283.3
Percent Broadleaf Forest Cover	1284.0
Null model	1286.5
